# Supplementary material for: An interplay between extracellular signalling and the dynamics of the exit from pluripotency drives cell fate decisions in mouse ES cells
Source: Biol Open. 2014 Jun 20;3(7):614–26. doi: 10.1242/bio.20148409 (PMC4154298; doi:10.1242/bio.20148409)
Supplement: Supplementary Material [file supp_3_7_614__index.html]

An interplay between extracellular signalling and the dynamics of the exit from pluripotency drives cell fate decisions in mouse ES cells — An interplay between extracellular signalling and the dynamics of the exit from pluripotency drives cell fate decisions in mouse ES cells — Supplementary Material 

# An interplay between extracellular signalling and the dynamics of the exit from pluripotency drives cell fate decisions in mouse ES cells

## bio.20148409 Supplementary Material

**Files in this Data Supplement:**

- Supplementary Material - David A. Turner et al. doi: 10.1242/bio.20148409
