# Supplementary material for: An interplay between extracellular signalling and the dynamics of the exit from pluripotency drives cell fate decisions in mouse ES cells
Source: Biol Open. 2014 Jun 20;3(7):614–26. doi: 10.1242/bio.20148409 (PMC4154298; doi:10.1242/bio.20148409)
Supplement: Supplementary Material [file supp_bio.20148409_bio.20148409-s1.pdf]

 Biology Open (2014) 000, 1–13 doi:10.1242/bio.20148409

Supplementary Material
David A. Turner et al. **doi: 10.1242/bio.20148409**

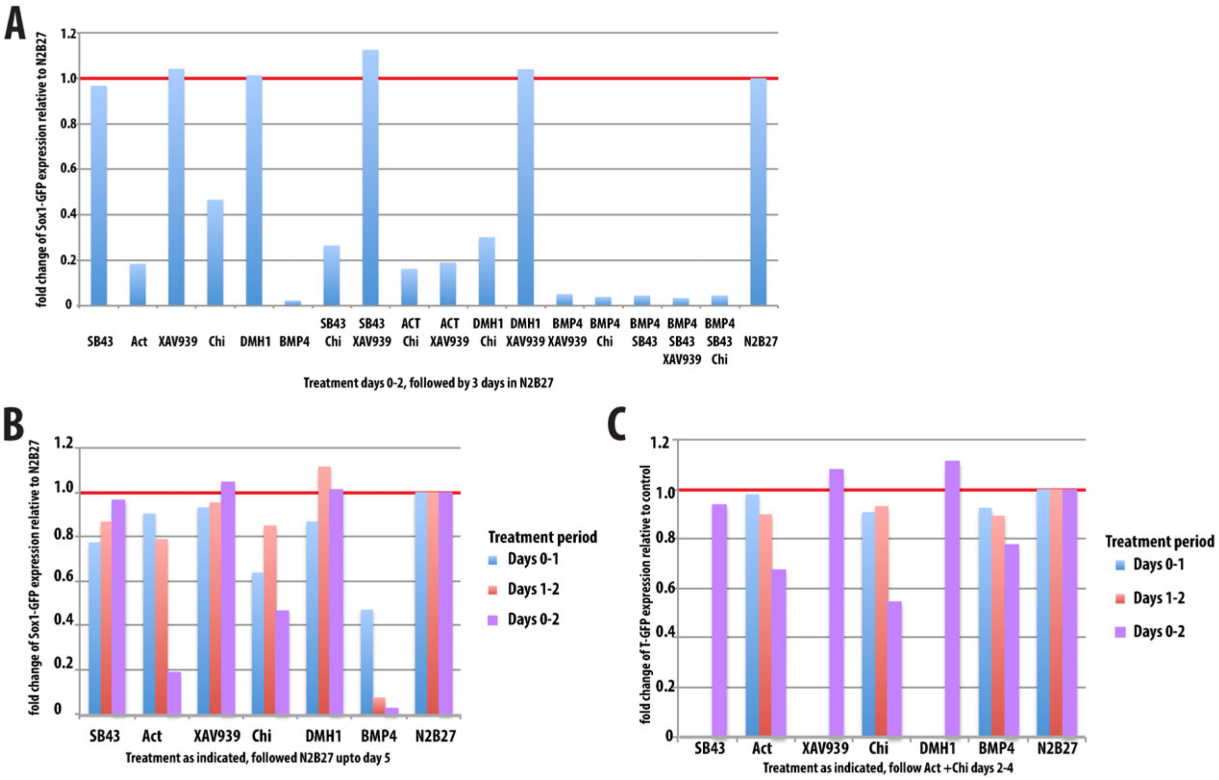

Fig. S1. Effects of signalling during the exit from pluripotency (supplementary data for Fig. 2). (A) Sox1::GFP cells were cultured in the indicated factors days 0–2, followed by days 2–5 in N2B27. (B,C) Sox1::GFP (B) or T::GFP(C) cells were maintained in N2B27 for 5 days (Sox1::GFP) or 2 days in N2B27, followed by 2 days in AC (T::GFP) and exposed to pulses of the treatment as indicated. GFP expression was analysed by flow cytometry and results were normalised to 5 days in N2B27 (Sox1::GFP) or 2 days N2B27 followed by 2 days AC (T::GFP). Note that the effect of the factors and treatments have remarkably similar effects on GFP expression in both Sox1::GFP cells and T::GFP cells.

Biology Open (2014) 000, 1–13 doi:10.1242/bio.20148409

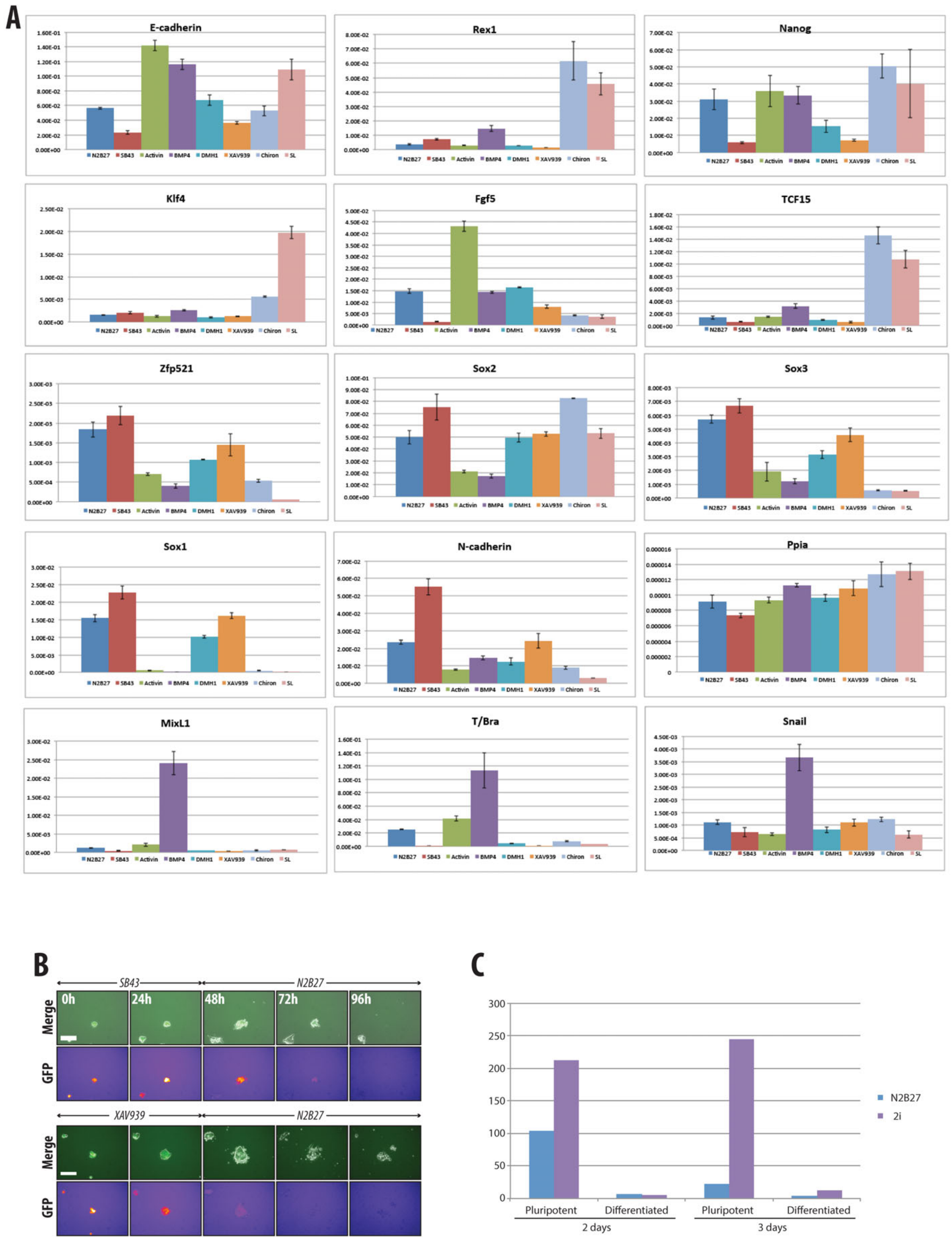

Fig. S2. See next page for legend.

Biology Open

Fig. S2. A fate restriction point at the exit from pluripotency (supplementary data for Fig. 3). (A) Sox1::GFP cells were grown in the indicated medium (ordinate) for 2 days and a further day in N2B27 prior to RNA extraction and RT-qPCR analysis for the indicated genes (graph titles). Data normalised to the house-keeping gene Ppia. (B) Live-cell imaging of TNGA Nanog::GFP cells treated with a 2-day pulse of either SB43 or XAV939 before wash-off and medium replacement with N2B27. (C) E14Tg2A mES cells maintained in S + L were plated into N2B27 or 2i as indicated for either 48 or 72 hours (2 or 3 days). Then 600 cells from each population were plated into 2i medium and cultured for a further 5 days; only cells that are pluripotent will form colonies. Cells initially plated in 2i for 2 days form 220 colonies, which increases to 257 colonies after 3 days in 2i. Whereas, cells initially plated into N2B27 for 2 days form 111 colonies, this declines to 27 colonies after 3 days in N2B27. Scale bars: 100 µm.

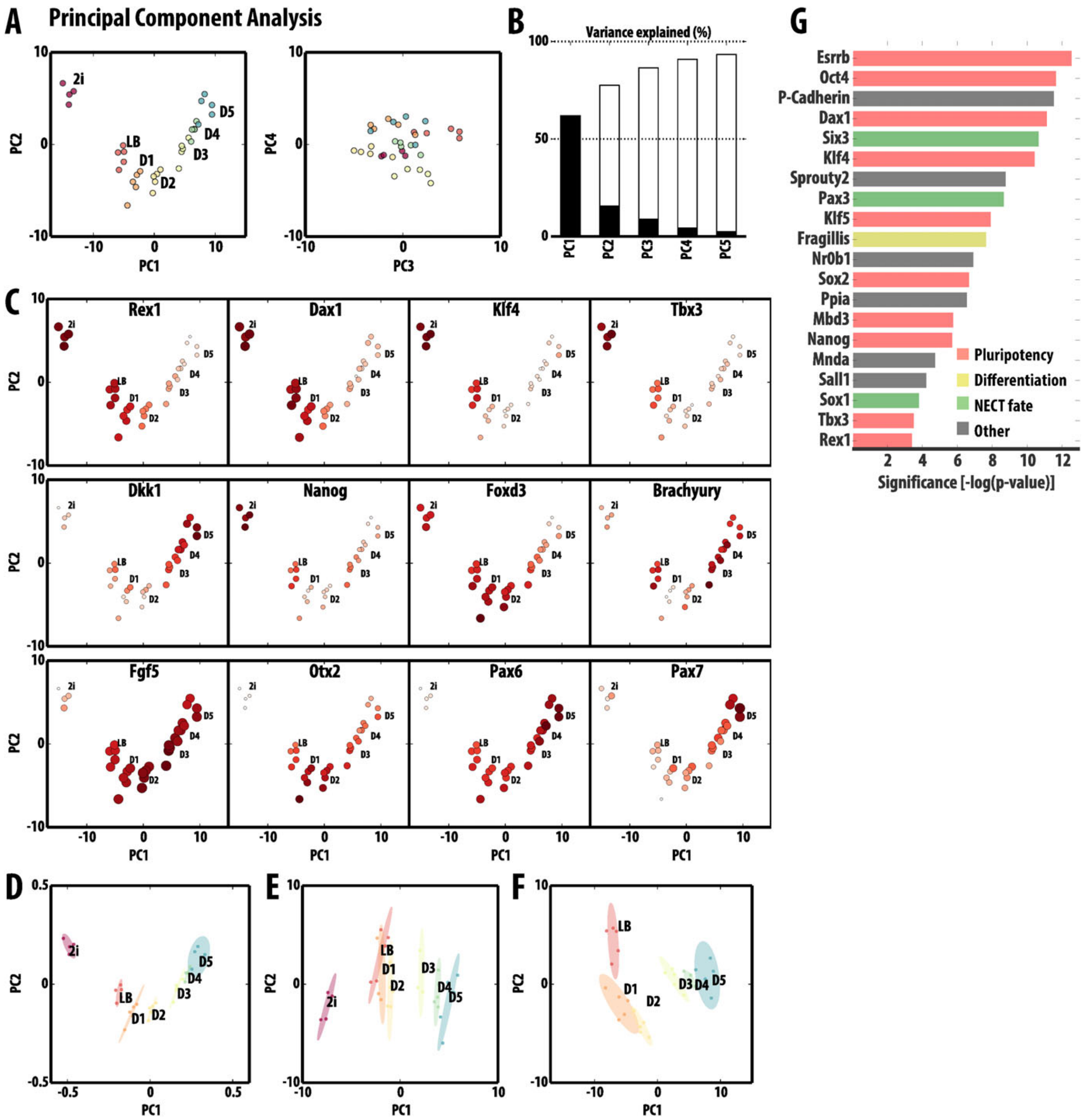

Fig. S3. Analysis of gene expression during the exit from pluripotency (supplementary data for Fig. 4). (A–F) Principal Component Analysis of bulk gene expression. We performed PCA of the gene expression profiles of 34 genes in 2i, BMP and LIF, and the 5-days differentiation in N2B27. The first four components are shown (A). Each dot corresponds to a technical repeat of a particular experimental condition (colour coded and labelled in the left panel). PC1 and PC2 cluster data according to each condition (A) and account for more than 75% of the total variance (B). (C) The levels of expression (coded in colour and size) of each condition for several genes of interest. (D,E) The results of the PCA are robust to data normalisation procedures. We obtained similar results doing PCA with data normalised with the min–max method (D) or the Z method (E). (F) The results of the PCA are hold when we do not consider the 2i conditions. (G) Single-cell gene expression analysis: top-most significant genes for differences in the distributions of expression levels of 4 days as well as the initial LIF and BMP condition (Kruskal–Wallis one-way test p-values corrected with Bonferroni for multiple testing).

Biology Open (2014) 000, 1–13 doi:10.1242/bio.20148409

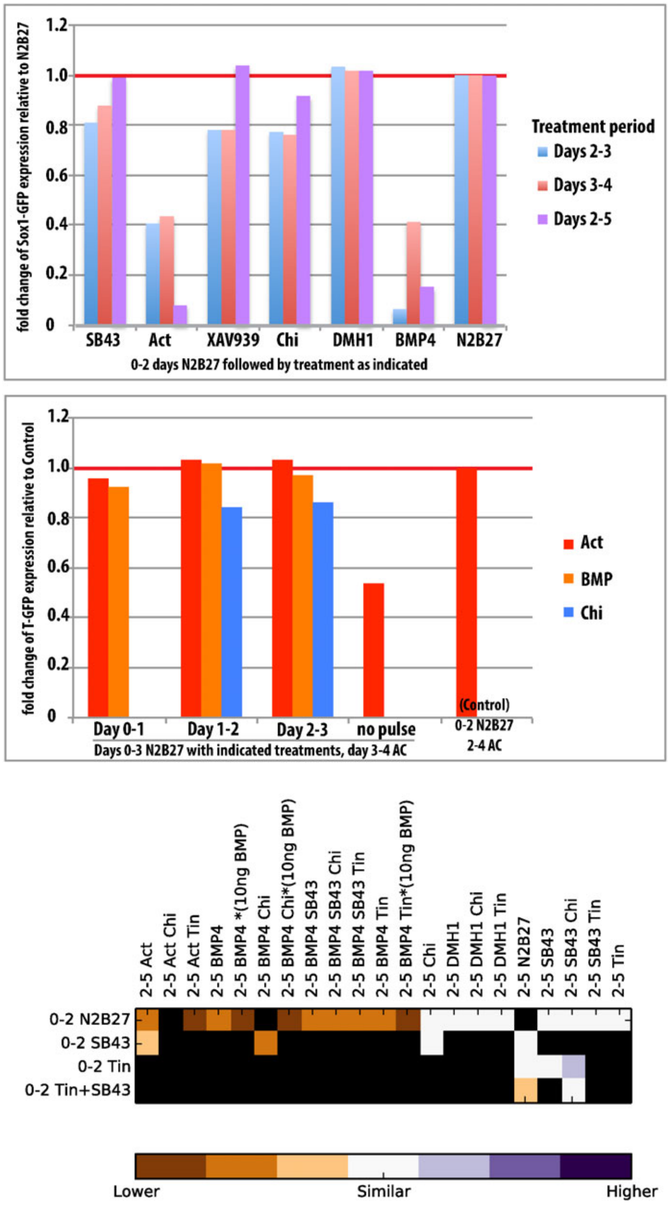

Fig. S4. Activin activity but not Wnt/β-Catenin activity controls fate decisions between PS and NECT (supplementary data for Fig. 5). Sox1::GFP cells were cultured in N2B27, SB43, XAV939 or SB43 + XAV939 for 0–2 days and, as indicated, days 2–5. GFP expression was analysed by flow cytometry, normalised to 5 days in N2B27 and is presented in the form of a heat map.

Biology Open

Biology Open (2014) 000, 1–13 doi:10.1242/bio.20148409

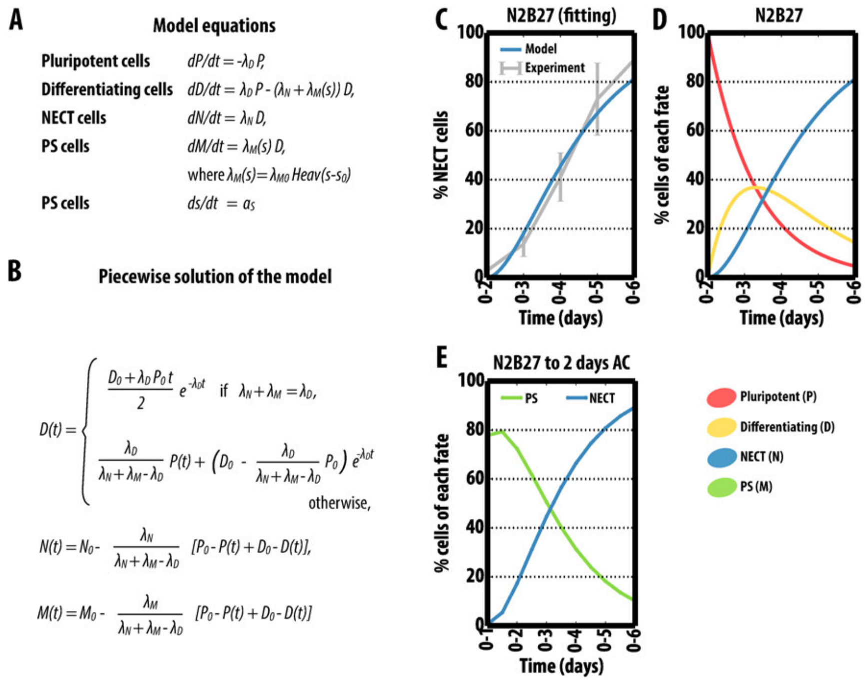

Fig. S5. A simple population model for a cell fate decision race (supplementary data for Fig. 6). (A) Model equations: the model consists of 4 different cellular types: pluripotent cells (P), differentiating cells (D), cells committed to NECT (N) and cells committed to PS (M). We consider initially all cells to be pluripotent. Cells then spontaneously lose pluripotency and start differentiating with a certain rate λ_D. Once each cell abandons the pluripotent state, rapidly and irreversibly adopts a final fate: either NECT or PS. Cells are assumed to acquire either fate at a particular rate: λ_N for NECT and λ_M for PS. In addition to these cellular types and the corresponding transitions, we also consider a signal (s) that builds up and once crosses a threshold, biases the rates of fate adoption. In particular, we consider that the rate of PS conversion is negligible in the absence of signal and becomes the largest rate once the signal concentration has reached the threshold, which we arbitrarily take to $s_0=1$. We initially consider all cells to be pluripotent ($P=1$, $D=N=M=0$). The model is linear but non-smooth, with piecewise constant $\lambda_M(s)$ depending on the signal level. The analytic solution of each region of constant λ_M is given in panel B. (C,D) Two of the three parameters (λ_D and λ_N) can be fitted to the experimental data on Sox1::GFP in N2B27 by assuming no cells adopt the PS fate ($\lambda_M=0$). (E) The model reproduces to a certain extent the 2-day Activin and Chiron pulse-chase experiment with the parameter values found in panel C and assuming a PS-fate adoption rate much higher than the NECT-fate adoption rate during the pulse (i.e. $\lambda_M >> \lambda_N$).
